# A cost-benefit analysis of genetic screening test for breast cancer in Iran

**DOI:** 10.1186/s12885-024-12003-4

**Published:** 2024-03-01

**Authors:** Zahra Meshkani, Najmeh Moradi, Ali Aboutorabi, Hiro Farabi, Nazi Moini

**Affiliations:** 1 Department of Health Economics, School of Health Management and Information Sciences, Iran University of Medical Sciences, Tehran, Iran; 2 Population Health Sciences Institute, Newcastle University, Newcastle upon Tyne, UK; 3 Department of Health Economics, School of Health Management and Information Sciences, Iran University of Medical Sciences, Tehran, Iran; 4 Barts and The London Pragmatic Clinical Trial Unit, Centre for Evaluation and Methods, Wolfson Institute of Population Health, Queen Mary University of London, London, UK; 5 Breast Cancer Research Centre, Motamed Cancer Institute, ACECR, Tehran, Iran; 6 Health Management and Economics Research Center, Iran University of Medical Sciences, 13833-19967 Tehran, Iran

**Keywords:** Breast cancer, BRCA1 protein, BRCA2 protein, Screening, Cost-benefit analysis, Willingness to pay, Economic evaluation

## Abstract

**Background:**

This study aimed to evaluate the implementation of the population- and family history (FH) -based screening for BReast CAncer (BRCA) in Iran, a country where less than 10% of breast cancer cases are attributable to a gene mutation.

**Methods:**

This was an economic evaluation study. The Benefit-Cost Ratio (BCR) for genetic screening test strategies in Iranian women older than 30 was calculated. To this end, the monetary value of the test was estimated using the willingness-to-pay (WTP) approach using the contingent valuation method (CVM) by payment card. From a healthcare perspective, direct medical and non-medical costs were considered and a decision model for the strategies was developed to simulate the costs. A one-way sensitivity analysis assessed the robustness of the analysis. The data were analyzed using Excel 2010.

**Results:**

660 women were included for estimating WTP and 2,176,919 women were considered in the costing model. The cost per genetic screening test for population- and FH-based strategies was $167 and $8, respectively. The monetary value of a genetic screening test was $20 and it was $27 for women with a family history or gene mutation in breast cancer. The BCR for population-based and FH-based screening strategies was 0.12 and 3.37, respectively. Sensitivity analyses confirmed the robustness of the results.

**Conclusions:**

This study recommends the implementation of a FH-based strategy instead of a population-based genetic screening strategy in Iran, although a cascade genetic screening test strategy should be evaluated in future studies.

## Introduction

Based on the GLOBOCAN estimate for 2020, the number of new cancer cases and deaths for both sexes and all age groups, excluding non-melanoma skin cancer, is estimated at 19.3 million cases and 10 million deaths worldwide, respectively. It is estimated that the incidence rate of cancer will increase to 47% (= 28.4 million new cases) in 2040 compared to 2020. Breast cancer was introduced as the most common type of cancer with 2.3 million new cases and a mortality rate of 6.9% [1, 2].

The incidence of breast cancer is different between countries. Developing countries have a higher mortality rate, while developed countries have a higher disease incidence. The age- standardized incidence rate in countries with a high and low human development index is 54.5 and 31.3 per 100,000 people, respectively [3].

Based on Chen et al., the global burden of cancer from 2020 to 2050 is $25.2 trillion, equivalent to an annual tax of 0.55% of global GDP, gross domestic product, and breast cancer was among the five cancers with the highest economic costs (= 7.7% of the global economic burden) [4]. Treatment costs for stages one to four of the disease in 2017 were approximately $283,000, $58,000, and $26,000 in North Carolina [5], Italy [6], and China [7], respectively. The direct medical costs for breast cancer and the average cost per patient in Papua New Guinea from 2017 to 2022 were $469,845.28 and $7248.47 respectively [8].

Overall, the treatment of cases, especially those at an advanced stage, is both financially and practically challenging. Early diagnosis of the disease to meet the challenges has been presented as the best strategy [9]. Although mammography is the common method in breast cancer screening and can reduce the risk of death by about 20% in women aged 50–59 years, especially in the early stages [10], a screening plan based on a woman’s risk of breast cancer has been recommended to be more effective [11].

Although demographic, breast-related, hormonal, reproductive, and lifestyle risk factors for breast cancer play a role in about 90% of cases, less than 10% of cases are due to hereditary and genetic mutations [12] which according to the WHO, they have a higher risk of developing breast cancer than others [13].

Although several genes (CHEK2, ATM, PALB2, and BRCA1/2) are attributed to breast cancer, the most important of these are the BRCA genes. The risk of developing breast cancer is on average 12% in the general population, but 65% in BRCA carriers [14–16]. BRCA carriers also have an increased risk of ovarian cancer. The risk of breast and ovarian is not invariant among carriers. Identifying BRCA carriers before they develop cancer is a success in prevention plans, and taking the time to perform preventive procedures such as risk-reducing salpingo-oophorectomy (RRSO) and risk-reducing mastectomy (RRM) to reduce the risk of disease [17].

Breast cancer is the most common in both sexes [18] and Tehran has the highest number of breast cancer in Iran [19]. The economic burden of breast cancer was estimated to be $193 million and $11,979 per patient in 2021 [20], and it is estimated that the number of new cases and the mortality rate of breast cancer will increase by 2035 [21]. The average age for breast cancer in Iranian women is 45 years, which is lower than in other countries [22, 23].

Because of the burden of the disease and the improvement of quality of life, policymakers would tend to prevent the disease, especially breast cancer as a disease with higher incidence [24] and it’s the goal of policymakers in Iran as well. Screening methods such as mammography are performed in Iran based on the WHO guidelines, while genetic screening tests for women at high risk, women with a gene mutation or a family history of breast or ovarian cancer, have recently started to be performed at the Genomic Research Centre of Iran. The tests are performed after pre-test counseling [25], and none of the recognized strategies, population-based and FH-based genetic screening tests, have been implemented in Iran.

The economic evaluation of new technologies could help policymakers allocate healthcare resources more efficiently and improve society’s quality of life. Cost-effectiveness studies (CEA) are used when QALY (quality adjusted life years) or DALY (disability-adjusted life years), as well as non-monetary outcomes, are important to choose the best intervention for a disease, while cost-benefit analysis (CBA) is used when the monetary outcome is more important for policymakers instead to budget allocation [26]. The WTP approach is a defined method to measure the benefits of interventions in health care systems [27, 28]. The cost-effectiveness of population-based versus FH-based genetic screening tests has been assessed in some studies, although most of them were conducted in high-income countries with Ashkenazi and Sephardic Jewish ethnicity and the cost-effectiveness of the population-based screening strategy has been confirmed [29–32], the present study aims to go a step further and evaluate whether Iran, as a low-income country where less than 10% of breast cancer cases attributable to a genetic mutation, is implementing BRCA screening test strategies, population-based or FH-based genetic screening tests.

## Methods

### Study design, study sampling

This was an economic evaluation study aimed at assessing the implementation of the two types of screening strategies, population-based and FH-based BRCA tests for early detection of breast cancer in Iran. To this end, two steps were carried out, estimating the monetary value and simulating the cost of the tests.

#### Step 1

The monetary value of the tests was estimated with the WTP approach using the CVM by payment card. Respondents were presented with a hypothetical scenario involving genetic screening tests and asked the following question.

“If the test were not free, what was the maximum amount you would be willing to pay out of pocket for a genetic test for breast cancer in the current year (2021)?”

All women older than 30 years in Tehran, the capital of Iran, in 2021 were included in this step because the relative risk of breast or ovarian cancer is high for BRCA carriers at this age [33]. The outcome of this step was the average monetary value of the genetic screening test for breast cancer. Further details of this step have been described in detail elsewhere [34].

#### Step 2

in this step, the direct medical and non-medical costs were considered from the perspective of the healthcare system. A decision analytic model for screening strategies was developed and the costs per screening for both strategies were simulated. The robustness of the analysis was evaluated using a sensitivity analysis.

The number of women over 30 years old in Tehran in 2021 was 2,791,909, based on the Statistical Centre of Iran [35]. The participation rate in genetic screening tests for breast cancer was assumed to be 71% based on Manchanda R et al. [30]. The decision analytic model, probabilities, and sensitivity analysis were explained in detail as follows.

### Genetic screening tests decision models

The Genome Research Centre in Iran followed NICE guidelines and offered BRCA genetic testing to women with a risk of ≥ 10% for the gene mutation. Population-based surveillance (every two-year mammogram between 40 and 60) was recommended for women with a negative BRCA mutation. For women with a positive BRCA mutation, RRM and RRSO are recommended initially, although not all eligible women undergo these procedures. Severe follow-up is recommended for women who have declined these procedures, including annual mammography and MRI at ages 40–70 and 30–50, respectively.

For premenopausal women included in our model, there is a risk of mortality from coronary heart disease (CHD) after RRSO, as there is with hormone replacement therapy (HRT). Breast prostheses were considered in women who had RRM.

**Fig. 1:**
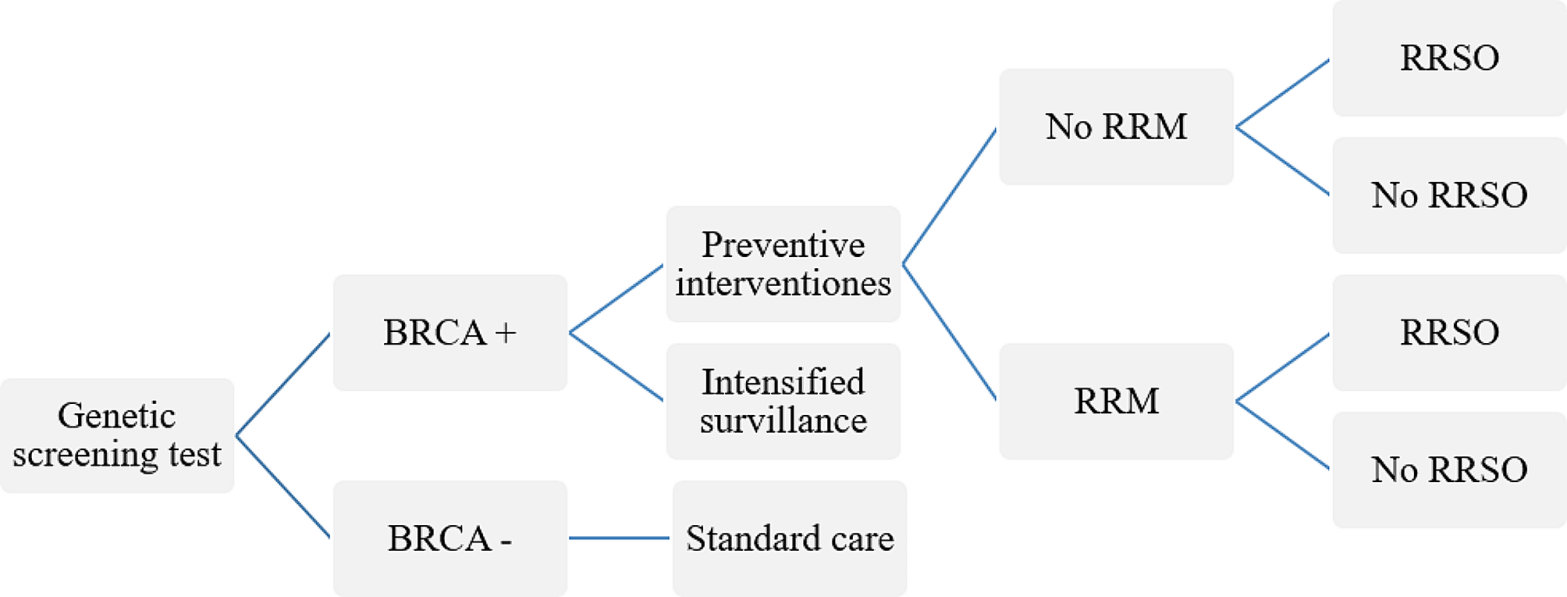
Decision model for population-based genetic screening BRCA tests

**Fig. 2:**
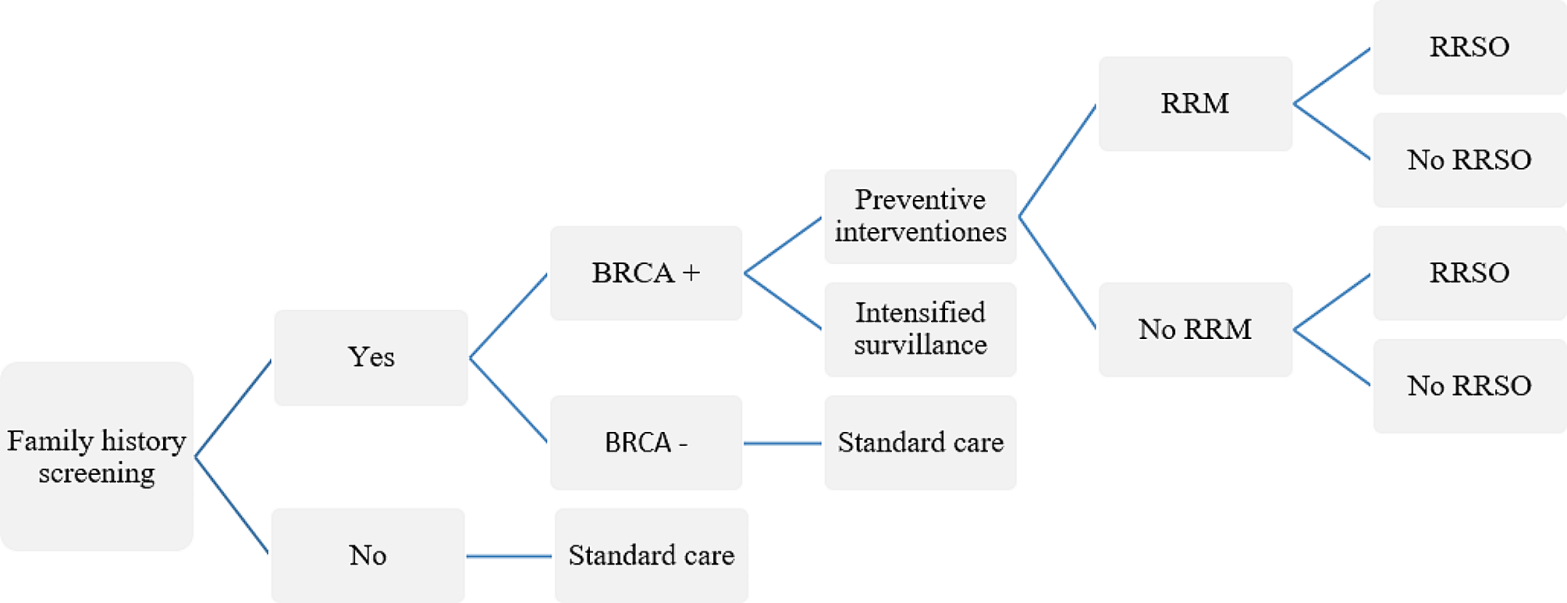
Decision model for FH-based genetic screening BRCA tests

In our study, the model for the population-based strategy assumes that all women older than 30 years are offered genetic testing, whereas, in the FH-based strategy, only high-risk women after genetic counseling, are offered genetic testing (Figs. 1 and 2).

### Probabilities

The probabilities used in our model based on the literature are described in Table 1. Since the studies had different probabilities, the probability-weighted averages corresponding to the sample size were used for the present study. Only the probabilities for BRCA mutations differed between the strategies.

**Table 1:** Probabilities used in the costing models

Description		Probability (%)	Sample size	Country	Probability-weighted Average (%)	References
BRCA1/BRCA2 mutation prevalence	BRCA1/BRCA2 mutation prevalence in a general population	0.890	2302	U.S.	0.5637	[36]
		0.392	5384	Malaysia		[37]
		0.677	1548	UK		[38]
	Probability of having a family history in the general population	0.98 (0.47–1.39)	NA	UK	0.98	[39]
	Probability of BRCA1/BRCA2 mutation in individuals with a family history of breast or ovary cancer	16.54	127	Greek	23.05	[40]
		19.14	418	Brazil		[41]
		23	133	China		[42]
		24	21,401	Germany		[43]
		21.50	349	Brazil		[44]
		23.05	256	North Spain		[45]
	BRCA1/BRCA2 mutation prevalence in breast cancer patients without a family history of breast cancer	11.06	434	Nigeria	10.47	[46]
		5	266	Peru		[47]
		13.43	134	Tunisia		[48]
		10.76	2769	China		[49]
Probability of preventive interventions	The probability that the carrier will undergo RRM	50	306	Denmark	26.4	[50]
		40	211	Manchester		[51]
		35.6	407	Netherland		[52]
		25.1	346	Slovenia		[53]
		18	1383	Canada		[54]
		21	325	Australia		[55]
	The probability that a BC patient will undergo RRM	33	581	Wales	31.5	[56]
		30	NA	UK		[57]
	The probability that a BRCA carrier will follow up with RRSO	74	305	North California	66	[58]
		58	170	New York		[59]
		52	42	Korea		[60]
		50	26	Netherland		[61]
	The probability that a BC patient will follow up with RRSO	37	581	Wales	59.23	[56]
		55	NA	UK		[39, 62]
		56.7	NA	UK		[29]
		66	NA	UK		[31]
	Performing MRI	30.6	1134	Canada	30.6	[54]
	Performing mammography	87.5	1134	Canada	87.5	[54]
Probability of side effects and other outcomes	Seroma	38.7	152	Iran	36.31	[63]
		32.8	103			[64]
	Breast prosthesis	87	148		74.51	[65]
		57.4	108			[66]
	Getting chronic disease because of oophorectomy	72 (68–76)	16,914	UK	72	[67]
	Performing hormone replacement therapy	39	75	Canada	37	[68]
		47	57	Netherland		[69]
		30	96	Canada		[70]

### Sensitivity analysis

One-way sensitivity analyses were conducted to check the robustness of the estimations. The minimum and maximum probabilities of the decision nodes were used for the analysis. The changes in probabilities were used separately for both strategies by considering general and high-risk women’s WTP.

### Data collection

#### For Step 1

, a population-based online survey was conducted from 4 July to 30 August 2021. A self-administered questionnaire was distributed via social media such as Telegram, WhatsApp, Instagram, and email invitations. Participants were free to answer the questionnaires. They were assured that their privacy would be respected.

The samples were selected through advertising on the pages and channels with the most members, randomly. The link to the questionnaire was also distributed to friends and colleagues.

#### For Step 2

, the costs of genetic counseling, BRCA genetic testing, RRM, breast prostheses, RRSO, CHD, bone health monitoring, HRT, mammography, and MRI were included as direct medical costs that were collected by the Iranian Ministry of Health based on Relative Value Unit (RVU) codes. Data on direct non-medical costs such as transportation and travel were requested from 90 cases, randomly.

### Data analysis

The BCR, the estimated monetary value divided by the cost per genetic screening test, was calculated. The monetary value per genetic screening test was considered for the population-based strategy while the monetary value per screening for high-risk women was considered for the FH-based screening strategy. If the result was greater than one, the defined strategy could recommended to be implemented. The data was analyzed using Excel 2010.

The following formula was used to simulate the cost of each strategy:

Total costs of strategies= ((average cost of genetic screening tests * targeted population based on probabilities) + (average cost of RRSO * targeted population based on probabilities) + (average cost of RRM * targeted population based on probabilities) + (average cost of side effects * targeted population based on probabilities) + (average cost of sever followed up * targeted population based on probabilities)) + (direct non-medical costs* targeted population based on probabilities).

## Results

1100 persons completed the questioner which after data cleaning, 660 women with an average age of 40 years were included for the estimation of WTP and 2,176,919 women for the cost model. The mean monetary value for the tests was $20, and the minimum and maximum values were $0.43 and $434, respectively. The monetary value for women with a family history of breast or ovarian cancer was 1.4 times (= $27) higher than for women without a family history of breast or ovarian cancer [34].

The costs of the genetic screening test were determined using decision models for both strategies. Genetic counseling and BRCA genetic screening tests were the most important cost drivers, averaging $177. RRM and RRSO were the recommended interventions for high-risk women, costing an average of $235 and $175, respectively. If women uptake these interventions, the costs of breast prostheses, CHD, and bone health monitoring were imposing as well, averaging $425, $348, and $8, respectively. Mammography and MRI, the costs averaged $24 were the cost drivers for low-risk as well as women who didn’t choose the preventive interventions. Descriptive statistics of the cost drivers are shown in Table 2.

**Table 2:** The descriptive statistics of genetic screening BRCA tests and associated costs

Intervention	Descriptive statistics	Costs ($US)
Genetic counseling and genetic screening BRCA tests (Number of cases = 33, Mean age = 41 years old)	Min	162
	Max	193
	Average	177
	STED.S	11
Risk-Reducing Mastectomy (RRM) (Number of cases = 234, Mean age = 50 years old)	Min	35
	Max	705
	Average	235
	STDVE.S	120
Breast prosthesis (Number of cases = 22, Mean age = 47 years old)	Min	69
	Max	824
	Average	425
	STDVE.S	333
Risk-Reducing Salpingo-Oophorectomy (RRSO) (Number of cases = 152, Mean age = 38 years old)	Min	20
	Max	636
	Average	175
	STDVE.S	104
CHD (Number of cases = 785, Mean age = 65 years old)	Min	107
	Max	723
	Average	348
	STDVE.S	166
Bone health monitoring (Number of cases = 580, Mean age = 58 years old)	Min	3
	Max	11
	Average	8
	STDVE.S	1
Mammography & MRI	Average	24

The result of cost simulation for population- and FH-based genetic screening tests is shown in Table 3. Tracing the resources and cost drivers showed that BRCA genetic screening tests accounted for more than 96% of the total costs for the population-based strategy. Preventive interventions (RRM & RRSO) accounted for less than 2% and direct non-medical costs accounted for less than 3%. In contrast, for the FH-based genetic screening strategy, the main cost drivers were direct non-medical costs and genetic counseling & genetic screening BRCA tests, accounting for 49.53% and 40.64% of total costs, respectively.

**Table 3:** The result of cost ($US) simulation of genetic screening tests for the genetic strategies

Cost items	Costs for population-based screening strategy	% of total cost	Costs for FH-based screening strategy	% of total cost
Genetic counseling and genetic screening BRCA tests	350,420,813	96.36	7,202,469	40.64
Preventive surgical interventions and associated costs	4,280,310	1.18	1,715,237	9.68
Preventive interventions (mammography and annual MRI)	68,157	0.02	27,312	0.15
Total direct medical costs	354,769,280	97.56	8,945,018	50.47
Total direct non-medical costs	8,881,488	2.44	8,777,327	49.53
Total direct medical and non-medical costs	363,650,768	100	17,722,345	100
Number of people entered into the models	2,176,919		2,176,919	
Number of high-risk individuals identified	12,271		4,917	
Cost per screening	167.05		8.14	

*US $1 in 2021 = 230,000 IRR

The cost per screening based on the population-based and FH-based genetic screening strategies was $167 and $8, respectively. Although the population-based genetic screening strategy had a higher cost per screening, it identified 12,271 high-risk women with breast and ovarian cancer, compared with 4917 high-risk women for the FH-based strategy.

The BCR for the FH-based screening strategy was 3.37, compared to 0.12 for the population-based screening strategy. Therefore, the FH-based genetic screening strategy is recommended.

The results were assessed based on a one-way sensitivity analysis of the maximum and minimum probabilities of the decision nodes and considering the WTP for genetic screening tests for women at general and high risk of breast cancer as well. The sensitivity analysis confirmed the robustness of the results and showed that the FH-based screening strategy had the highest BCR compared to the population-based screening strategy.

## Discussion

Iranian health policymakers are focusing more on the prevention and treatment of breast cancer, as this is the most common type of cancer, and the incidence of the disease is also increasing. In Tehran, there are more than 2500 thousand women older than 30 years and policymakers tend to prevent breast cancer as it is a high economic burden and quality of life. Therefore, evaluating screening strategies helps policymakers to early diagnose the disease and save costs. While around 10% of breast cancer cases are attributed to a family history of breast or ovarian cancer and a genetic mutation, only mammography is performed in Iran. As a result, the goal of screening is not achieved and high-risk women develop advanced stages of breast cancer, which also leads to high treatment costs.

On average, treatment costs for stage I to IV breast cancer in 2015 were $29,724 $39,322, $57,828, and $62,108 respectively [71] and it is $2870.08, $6938.57, $ 9973.76, and $ 14105.73 in Iran [20]. Due to an efficient screening strategy, the present study focused on the economic evaluation of genetic screening test strategies (population- and FH-based screening).

Based on the results, the costs per screening for population-based and FH-based screening are $167 and $8, respectively and by $20 WTP, The BCR for the FH-based screening strategy was 3.37 and for the population-based screening strategy 0.12. Therefore, FH-based screening was recommended in Iran.

Studies on the economic evaluation of genetic screening tests are limited. Based on a cost-effectiveness study by Manchanda et al., population-based genetic screening tests for women older than 30 years were cost-saving from a social perspective in high-income countries (including the United States, the United Kingdom, and the Netherlands), while they were extremely cost-effective in high- and middle-income countries (including Brazil and China) and not cost-effective in low-income countries (including India) due to the cost of genetic screening tests [39]. According to our results, BRCA genetic testing accounted for more than 96% of total costs in the population-based strategy, compared with only about 41% in the FH-based strategy. The cost of the tests plays an important role in the choice of strategy. Genetic screening tests are a new technology in Iran and their costs are high. Willingness to pay for testing as a benefit to cover testing costs is critical to implementing a screening strategy. The results of the present study showed that the benefits of genetic screening strategies are on average $20 by the WTP approach and more than 80% of Iranian women had intention to do the genetic screening tests. Iranian women do not have enough knowledge about the benefits of the tests, especially about assessing the risk of family members for breast and ovarian cancer. BRCA carriers have a risk of ovarian cancer as well [17]. The results of Guo et al. [72] were the same and based on the study most Hispanic women (in a low-income country), have $25 WTP for genetic tests. It is interesting that they didn’t like to do preventive interventions such as RRM and RRSO and have poor knowledge about genetic screening tests for breast cancer such as Iranian women. Shame was an important factor that led to the postponement of the screening strategies for breast cancer by Iranian women [73] therefore paying attention to physiological factors and improving women’s knowledge as well as their awareness about the benefits of screening could help them participate in preventive intervention plans.

Another study by Manchanda et al. looking at genetic screening BRCA tests in Ashkenazi women and women at high-risk for breast cancer found that genetic screening tests were cost-effective for all women over 35 years of age, while FH-based screening was not cost-effective because some high-risk women were not identified by the strategy. Consequently, family history was not recommended [74], which was in contrast to our study. In our study, FH-based genetic screening tests are recommended, although a population-based strategy would identify about 2.5 times more high-risk women with breast cancer. The reason for the difference between the results lies in the objective and perspective of the studies. Outcome and cost perspectives are important factors in economic evaluation studies that can change the results. The focus of our study as a cost-benefit evaluation was on the monetary outcome of genetic testing through the WTP approach to estimate the budget required for screening strategies. The setting of the studies is another difference factor between the studies. Iran is a country with less than 10% genetic breast cancer, and more than 90% of breast cancer cases are not attributed to a genetic mutation. Therefore, it is not logical to allocate a high budget for a population-based screening strategy but Ashkenazi women are at high-risk for a gene mutation. Since mammography is the current screening strategy, the economic evaluation in Iran is limited to the strategy. It is an important point that genetic screening tests are a parallel strategy to mammography, and it is recommended for policymakers to use them for efficient screening. Therefore, the evaluation of mammography screening and genetic screening tests should be assessed as well. The study by Hatam et al. found that mass screening of mammography has higher costs compared to no-screening and was not recommended for all women over 25 years of age [75]. The study by Schousboe et al. found that biennial mammography screening up to the age of 80 years is a cost-effective option [76]. Mammography and genetic screening tests have different time horizons. It is sufficient to do the test only once in a lifetime and its benefits will accumulate in the year of performing the screening, but mammography is performed every two years for high-risk people and annually for low-risk people over 40 years.

Overall, genetic screening testing can identify high-risk women with breast cancer as well as ovarian cancer lead to managing the disease (determining the type and interval of breast cancer screening or deciding to perform preventive interventions) and cost savings as well as efficient healthcare budgeting. To have a comprehensive view, the prevalence of breast cancer, the number of women older than 30 years, the physiological factors for women’s uptake of screening tests, income, and insurance coverage should be considered for the success of a screening plan.

Although to our knowledge this is the first study to examine the cost-benefit effect of a genetic screening test in terms of its implementation to help policymakers choose the best strategy to improve quality of life while reducing healthcare costs, an in-person interview to ask about the direct non-medical costs was not possible due to the COVID-19 pandemic, so cases were invited to participate in a telephone interview. We did not include intangible costs in the cost calculation models.

## Conclusion

The present study recommended the implementation of a FH-based instead of a population-based genetic screening strategy in Iran. It is suggested that the economic evaluation of the cascade screening strategy be considered in future studies. It is important to say that genetic screening tests are performed in parallel with mammography. The genetic screening tests could contribute to cost savings as women at high risk of ovarian cancer are identified as well.

## Data Availability

All data generated or analyzed during this study are included in this published article.

## Abbreviations

BRCA: BReast CAncer
FH-based screening: Family History bases screening
BCR: Benefit-Cost Ratio
WTP: Willingness-To-Pay
CVM: Contingent Valuation Method
GDP: Gross domestic product
RRSO: Risk-Reducing Salpingo-Oophorectomy
RRM: Risk-Reducing Mastectomy
QALY: Quality Adjusted Life Years
CEA: Cost-effectiveness studies
DALY: Disability-Adjusted Life Years
CBA: Cost-Benefit Analysis
CHD: Coronary Heart Disease
HRT: Hormone Replacement Therapy
RVU: Relative Value Unit
